# Protective Effect of Hainosankyuto, a Traditional Japanese Medicine, on *Streptococcus pyogenes* Infection in Murine Model

**DOI:** 10.1371/journal.pone.0022188

**Published:** 2011-07-22

**Authors:** Masaaki Minami, Mariko Ichikawa, Nanako Hata, Tadao Hasegawa

**Affiliations:** 1 Department of Bacteriology, Nagoya City University Graduate School of Medical Sciences, Nagoya, Japan; 2 Department of Microbiology, Nagoya City University Hospital, Nagoya, Japan; Statens Serum Institute, Denmark

## Abstract

**Background:**

*Streptococcus pyogenes* (*S. pyogenes*) causes various serious diseases including necrotizing fasciitis and streptococcal toxic shock syndrome. One serious problem observed recently with *S. pyogenes* therapy is attenuation of the antibiotic effect, especially penicillin treatment failure and macrolide resistance. Hainosankyuto, a traditional Japanese medicine based on ancient Chinese medicine, has been used for treatment of infectious purulent diseases in Japan. In this study, we investigated the protective and therapeutic efficacy of Hainosankyuto against *S. pyogenes*-skin infection.

**Methodology/Principal Findings:**

A broth microdilution method revealed that Hainosankyuto did not show a direct anti-bacterial effect against *S. pyogenes*. Force-feeding Hainosankyuto to infected mice for 4 consecutive days increased the survival rate and reduced the size of local skin lesions compared with mice fed PBS. Although we did not find the significant recovery of survival rate in Hainosankyuto administration only after *S. pyogenes* infection, the sizes of ulcer lesion were significant smaller after Hainosankyuto administration compared with mice fed PBS. No difference was observed in the anti-bacterial effect of Hainosankyuto between macrolide-susceptible and -resistant strains. Blood bactericidal assay showed that the survival rate of *S. pyogenes* using the blood from Hainosankyuto -treated mice was lower than that using the blood from untreated mice. We also found increased levels of IL-12, IFN-γ and a decreased level of TNF-α in the serum of *S. pyogenes*-infected mice treated with Hainosankyuto. Mouse peritoneal macrophage from Hainosankyuto-treated mice had significant phagocytic activity and increased mRNA levels of IL-12, IFN-γ and decreased mRNA level of TNF-α compared with control macrophage.

**Conclusions/Significance:**

Hainosankyuto increased survival rate after *S. pyogenes* infection and up-regulated both blood bactericidal activity and macrophage phagocytic activity through modulation of inflammatory cytokines. Our data also suggest Hainosankyuto may be useful for the treatment of *S. pyogenes* infection more prophylactically than therapeutically.

## Introduction

The gram-positive bacterium *Streptococcus pyogenes* (*S. pyogenes*) is the most common agent causing upper respiratory tract and skin infections [1]. It is also responsible for post-streptococcal diseases, such as rheumatic fever and glomerulonephritis, in addition to increasing the incidence of invasive infections such as streptococcal toxic shock syndrome [1].

One serious problem with *S. pyogenes* therapy is attenuation of the antibiotic effect. Despite the fact that *S. pyogenes* is always susceptible to penicillin, *S. pyogenes* infects up to 20% of penicillin-treated patients, and half of these cases are also clinical failures [2]. In recent years, macrolide- and lincosamide-resistant *S. pyogenes* isolates have gradually spread worldwide [3]. Novel anti-microbial agents other than antibiotics are now being sought.

Traditional herbal medicines are being re-evaluated in the clinical field because of their relatively few side effects and suitability for long-term administration compared with synthetic medicines. Recently, several types of these herbal medicines have been used clinically for various diseases in Japan [4]. Previous reports have described the biological actions of herbal medicines on immune responses in bacterial infection [5]. Herbal medicine research determining the role of entomopathogenic fungus *Cordyceps sinensis* (*C. sinensis*) in anti-*S. pyogenes* therapy has been reported [6]. Production of cytokines, such as interleukin (IL)-12, interferon (IFN)-γ and tumour necrosis factor (TNF)-α , and upregulation of phagocytic activity by the *C. sinensis* fraction might play roles in anti-tumour and anti-bacterial activities.

Hainosankyuto, a traditional herbal Japanese medicine based on ancient Chinese medicine, has been used for the treatment of infectious purulent diseases and has been suggested to have various functions. However, to date Hainosankyuto has not been investigated for its role in anti-bacterial therapy.

In the present study, we evaluated the protective efficacy of Hainosankyuto against *S. pyogenes* using a murine skin infection model. We pre-treated ICR mice with either Hainosankyuto or PBS and administrated Hainosankyuto or PBS after *S. pyogenes* infection for 2 days. We then examined serum cytokine levels, blood bactericidal activity and mortality in both groups of mice at each day. Furthermore we also evaluated the therapeutic effect of Hainosankyuto against *S. pyogenes* infection and the phagocytic activity of Hainosankyuto-treated mouse macrophage as the mechanism of protection.

## Results

### MIC determination

MIC of Hainosankyuto was above 256 µg/mL. No anti-bacterial effect of Hainosankyuto against *S. pyogenes* was observed.

### Murine infection model

**Figure 1:**
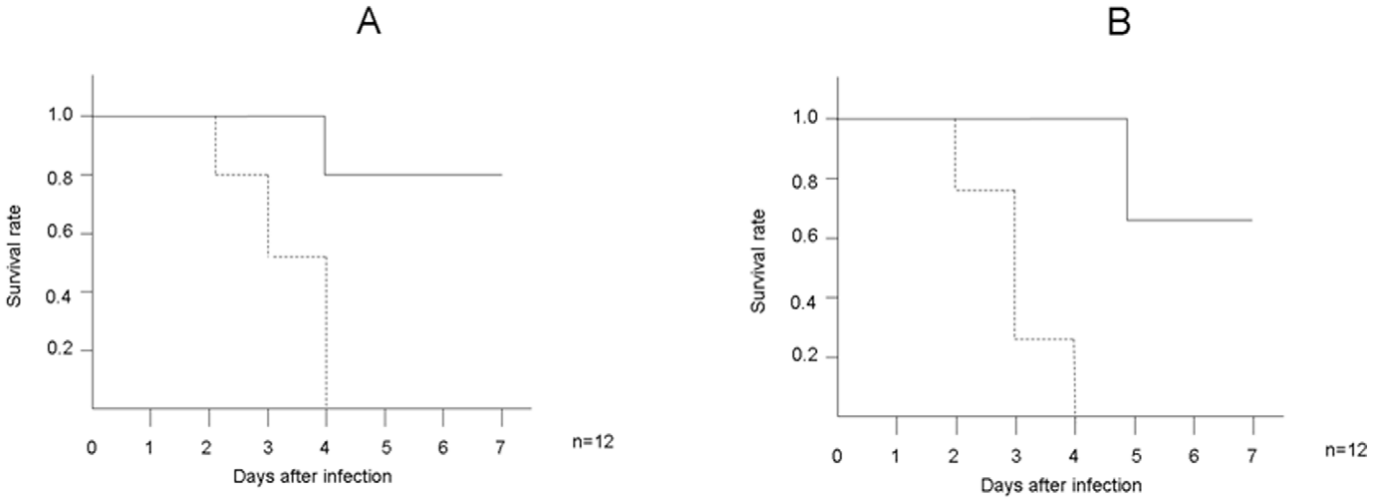
Prophylactic administration of Hainosankyuto increased the survival rate of *S. pyogenes* -infected murine models. Three-week-old ICR mice were force-fed Hainosankyuto ( ) or PBS ( ) for 4 consecutive days (day −1, 0, 1, and 2) and inoculated with 1×10^8^ CFU of *S. pyogenes* 1529 (Figure 1A) and D2TY (Figure 1B) at day 0. Mortality was monitored for 7 days. Survival data were assessed by Kaplan–Meier survival analysis and tested for significance using the log-rank test.

**Figure 2:**
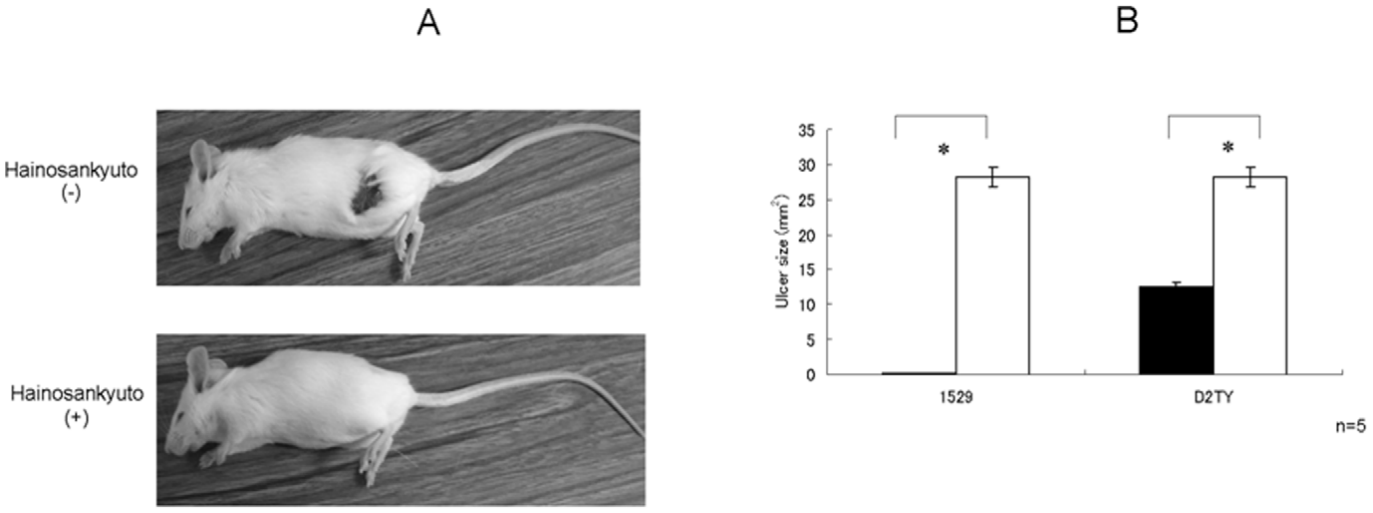
Prophylactic administration of Hainosankyuto prevented the ulcer in *S. pyogenes* 1529 and D2TY -infected murine models. Three-week-old ICR mice were subcutaneously inoculated with 1×10^8^ CFU of *S. pyogenes* 1529 (Figure 2A). Mice were observed daily for lesion and necrosis formation. Hainosankyuto decreased the size of ulcerative lesions in *S. pyogenes*-infected murine model (Figure 2B). Lesion size (length×width) was measured with length determined as the longest dimension of the lesion. Closed circle and open circle represented Hainosankyuto -treated and -untreated mouse, respectively. Lesion size data were tested for significance using the *t*-test. *: *p*<0.05.

In the present study, an animal model was used to test whether Hainosankyuto would provide the host with a protective effect against *S. pyogenes* infection. Mice in the control group started to die on day 2 after inoculation with *S. pyogenes* 1529, and the survival rate dropped to 0% after day 4. Mice in the Hainosankyuto-treated group started to die on day 4 after inoculation with *S. pyogenes* 1529, and the survival rate remained at 80% after day 7 (Figure 1A). In addition, mice in the control group started to die on day 2 after inoculation with *S. pyogenes* D2TY, and the survival rate dropped to 0% after day 4. Mice in the Hainosankyuto-treated group started to die on day 5 after inoculation with *S. pyogenes* D2TY, and the survival rate remained at 60% after day 7 (Figure 1B). Furthermore, as shown in Figure 2A, lesion size on day 3 after injection was smaller in the Hainosankyuto-treated mice injected with *S. pyogenes* 1529 compared to the control mice (*p*<0.05). Lesion size in the *S. pyogenes*-infected mice was measured, and Figure 2B represented the significant difference observed in lesion size between Hainosankyuto-treated and untreated mice (*p*<0.05).

**Figure 3:**
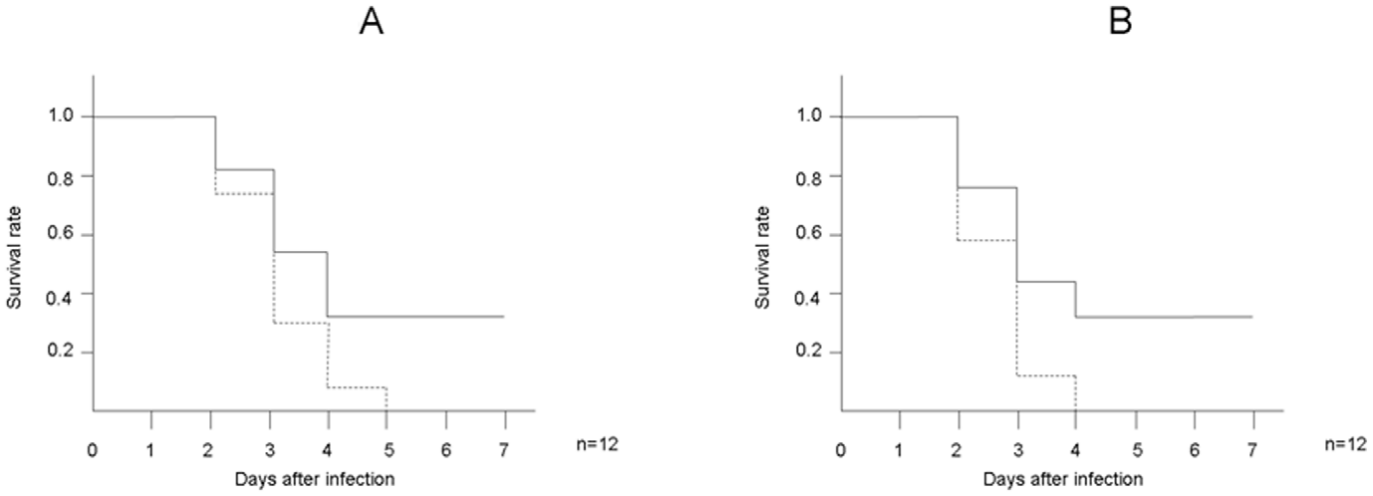
Therapeutical administration of Hainosankyuto tended to increase the survival rate of *S. pyogenes* -infected murine models. Three-week-old ICR mice were inoculated with 1×10^8^ CFU of *S. pyogenes* 1529 (Figure 3A) and D2TY (Figure 3B). After 24 hour, they were force-fed Hainosankyuto ( ) or PBS ( ) for 4 consecutive days (day 1, 2, 3, and 4) and. Mortality was monitored for 7 days after infection. Survival data were assessed by Kaplan–Meier survival analysis and tested for significance using the log-rank test.

**Figure 4:**
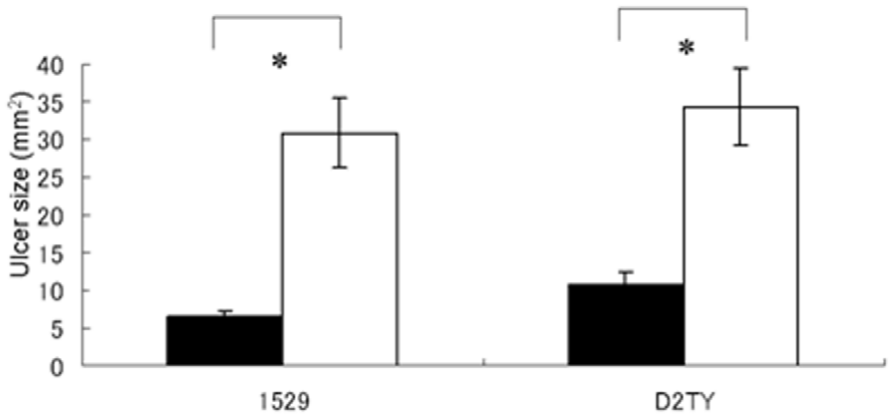
Therapeutical administration of Hainosankyuto prevented the ulcer in *S. pyogenes* infected murine model. Three-week-old ICR mice were subcutaneously inoculated with each strain. Mice were observed daily for lesion and necrosis formation. Lesion size (length×width) was measured with length determined as the longest dimension of the lesion. Closed circle and open circle represented Hainosankyuto -treated and -untreated mouse, respectively. Lesion size data were tested for significance using the t-test. *: *p*<0.05.

We next investigated whether Hainosankyuto was useful therapeutically. Mice in the Hainosankyuto-treated group started to die on day 2 after inoculation with *S. pyogenes* 1529, and the survival rate remained at 33% after day 7 (*p* = 0.0589)(Figure 3A). In addition, mice in the control group started to die on day 2 after inoculation with *S. pyogenes* D2TY, and the survival rate dropped to 0% after day 4. Mice in the Hainosankyuto-treated group started to die on day 2 after inoculation with *S. pyogenes* D2TY, and the survival rate remained at 33% after day 7 (*p* = 0.0569) (Figure 3B).Although the survival rates of Hainosankyuto-treated group were higher than those of control group, there was no significant differences between these 2 groups. However, lesion size in the *S. pyogenes*-infected mice was measured, and Figure 4 demonstrated the significant difference observed in lesion size between Hainosankyuto-treated and untreated mice (*p*<0.05).

### Blood bactericidal assays

**Figure 5:**
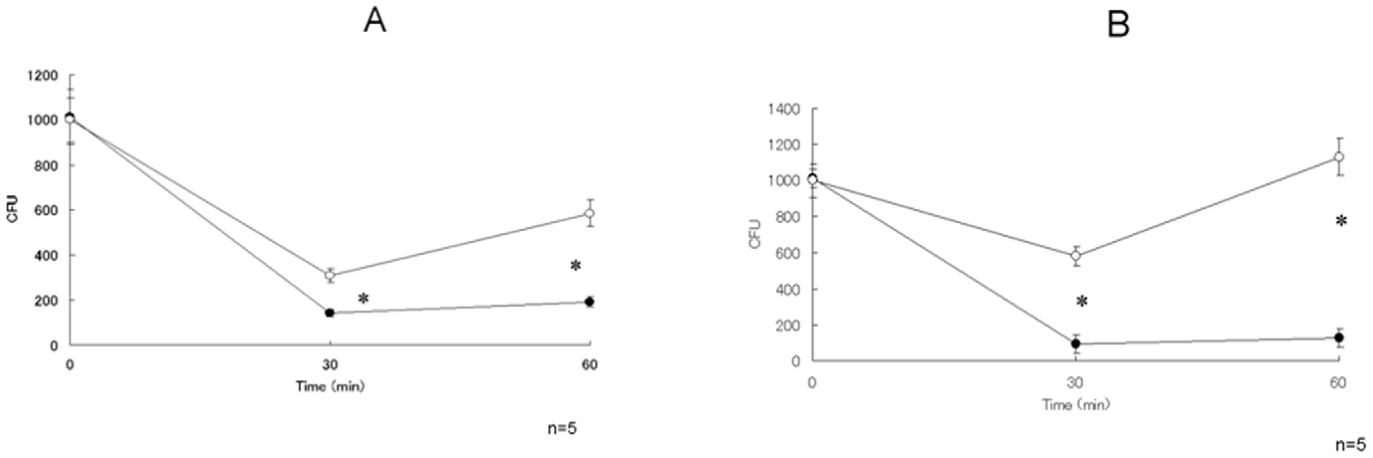
Blood bactericidal assay in *S. pyogenes*-infected murine models. Approximately 1000 CFU of *S. pyogenes* 1529 (Figure 5A) and D2TY (Figure 5B) were added to 1 ml of heparinised 3-week-old ICR mouse whole blood and rotated at 37°C. Diluted samples of blood were plated at the indicated time points to determine the number of CFU. Closed circle and open circle represented Hainosankyuto -treated and -untreated mouse, respectively. *: *p*<0.05.

To determine whether leukocytes in Hainosankyuto-treated mice show elevated bactericidal activity, we performed *S. pyogenes* killing assays using mouse blood. As shown in Figure 5A, blood from untreated mice killed approximately 70% of the bacterial inoculum after a 30-min incubation at 37°C. Similar levels of *S. pyogenes* killing were observed in the blood from Balb/C mice (data not shown). The blood from Hainosankyuto-treated mice killed approximately 90% of *S. pyogenes* inoculum after a 30-min incubation at 37°C. Significant differences were observed in *S. pyogenes* 1529 CFU reduction between Hainosankyuto-treated and untreated mice (*p*<0.05) (Figure 5A). Significant differences were observed in *S. pyogenes* D2TY CFU reduction between Hainosankyuto-treated and untreated mice (*p*<0.05) (Figure 5B).

### Analysis of serum cytokines during infection

**Figure 6:**
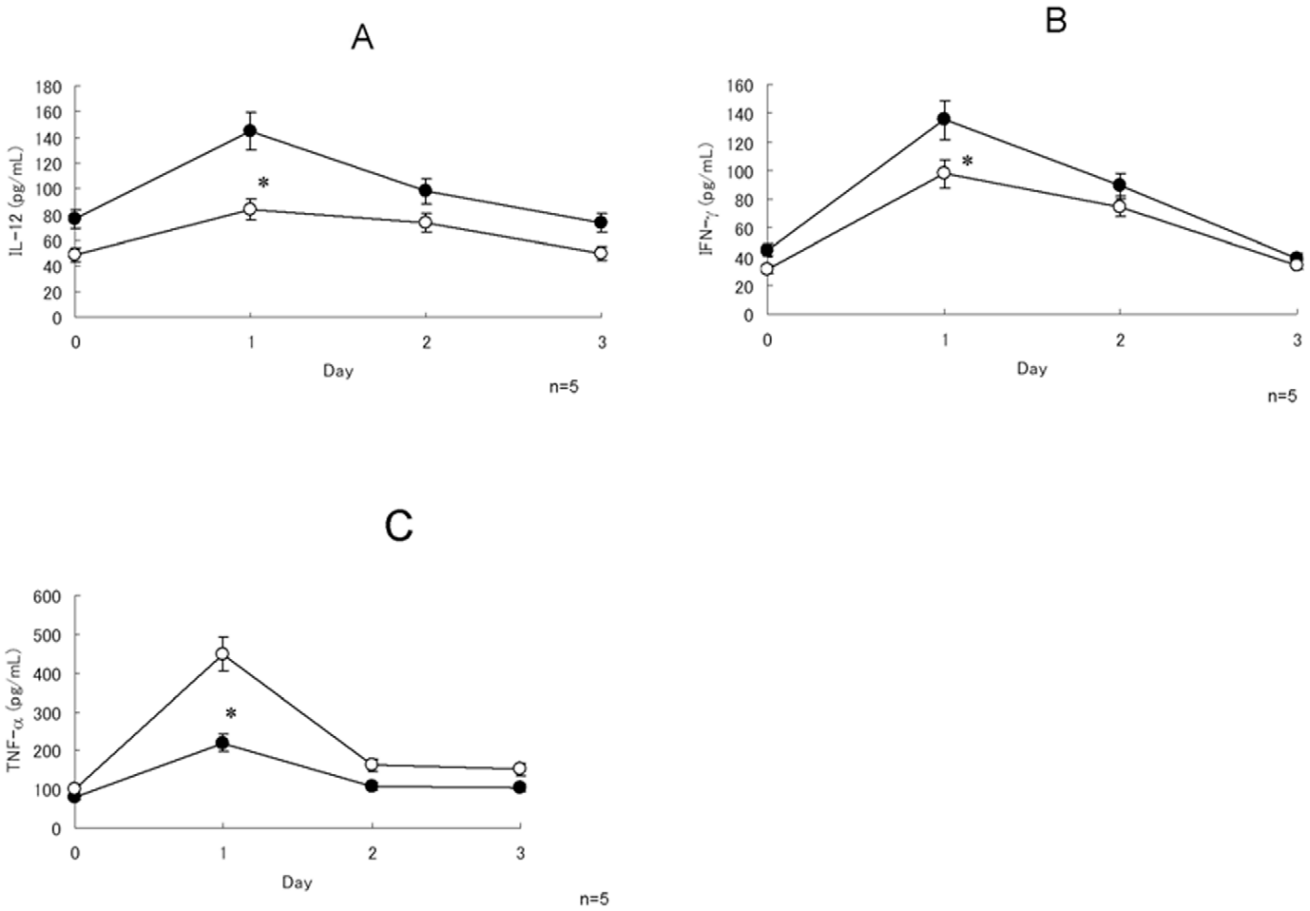
Time course of serum IL-12, IFN-γ and TNF-α levels determined by ELISA in *S. pyogenes*-infection. Three-week-old ICR mice were given 1 g/kg body weight of Hainosankyuto or PBS before infection. After *S. pyogenes* 1529 infection, mice were sacrificed under CO_2_ anaesthesia at adequate time and blood samples were collected. Serum IL-12 (Figure 6A), IFN-γ (Figure 6B), and TNF-α (Figure 6C) were analyzed by ERISA. Closed circle and open circle represented Hainosankyuto -treated and -untreated mouse, respectively. *: *p*<0.05.

The kinetics of appearance of cytokines, such as IL-12, IFN-γ and TNF-α, the serum of infected mice with or without Hainosankyuto treatment was investigated. Mice in both groups showed significantly elevated serum IL-12 levels when compared to those before injection (*p*<0.05) (Figure 6A). Mice in the Hainosankyuto-treated group showed a significantly elevated serum IL-12 and IFN-γ level compared to that in control mice (*p*<0.05) (Figure 6B). A significant decrease in the TNF-α level was observed in the Hainosankyuto-treated group at day 1 (*p*<0.05) (Figure 6C). However, no significant difference was observed in TNF-α level between control and infected groups on days 0, 2 and 3.

### Effect of Hainosankyuto on phagocytic activity

**Figure 7:**
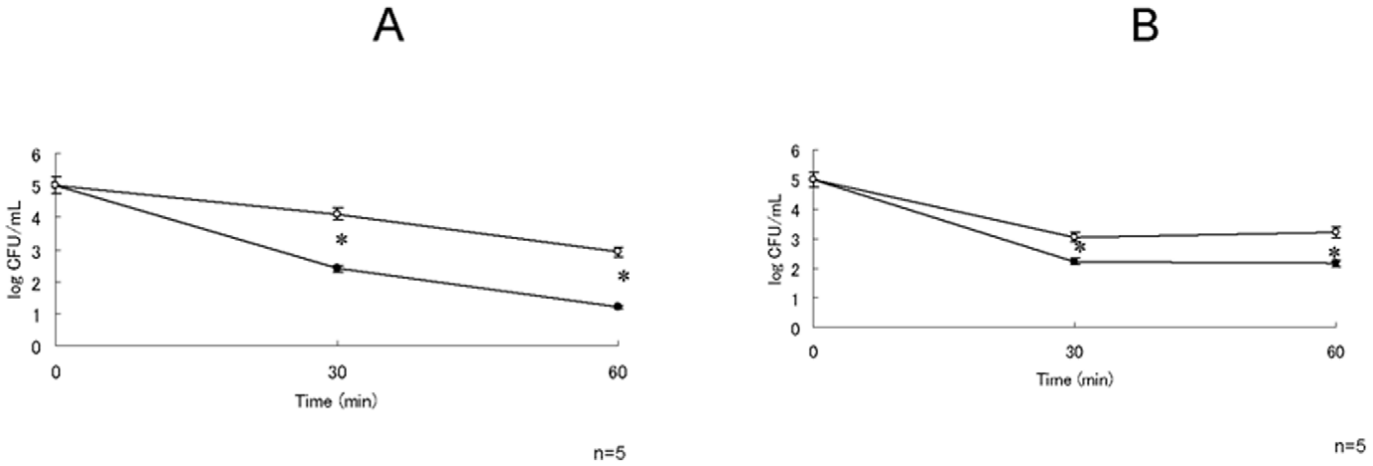
Mouse peritoneal macrophage phagocyte assay. Three-week-old ICR mouse peritoneal macrophage were prepared and determined by mixing cells with *S. pyogenes* 1529 (Figure 7A) or D2TY (Figure 7B) at 37°C. Diluted samples were plated at the indicated time points to determine the number of CFU. Closed circle and open circle represented Hainosankyuto -treated and -untreated mouse, respectively. *: *p*<0.05.

We next focussed on macrophage activity from leukocytes because macrophage plays a major role in phagocytic activity in murine infection model. To determine whether peritoneal macrophage from Hainosankyuto-treated mice show elevated phagocytic activity, we performed phagocytic assays on *S. pyogenes*. As shown in Figure 7A, macrophage from untreated mice killed approximately 60% of the *S. pyogenes* 1529 inoculum after a 30-min incubation at 37°C. The macrophage from Hainosankyuto-treated mice killed more than 99% of *S. pyogenes* 1529 inoculum after a 30-min incubation at 37°C. Figure 7B showed that macrophage from untreated mice killed approximately 90% of the *S. pyogenes* D2TY inoculum after a 30-min incubation at 37°C. The macrophage from Hainosankyuto-treated mice killed more than 99% of *S. pyogenes* D2TY inoculum after a 30-min incubation at 37°C. Significant differences were observed in both *S. pyogenes* 1529 and D2TY CFU reduction between Hainosankyuto-treated and untreated mice (*p*<0.05) (Figure 7A, Figure 7B).

### Cytokine expression of perotineal macrophage in Hainosankyuto-treated mice

**Figure 8:**
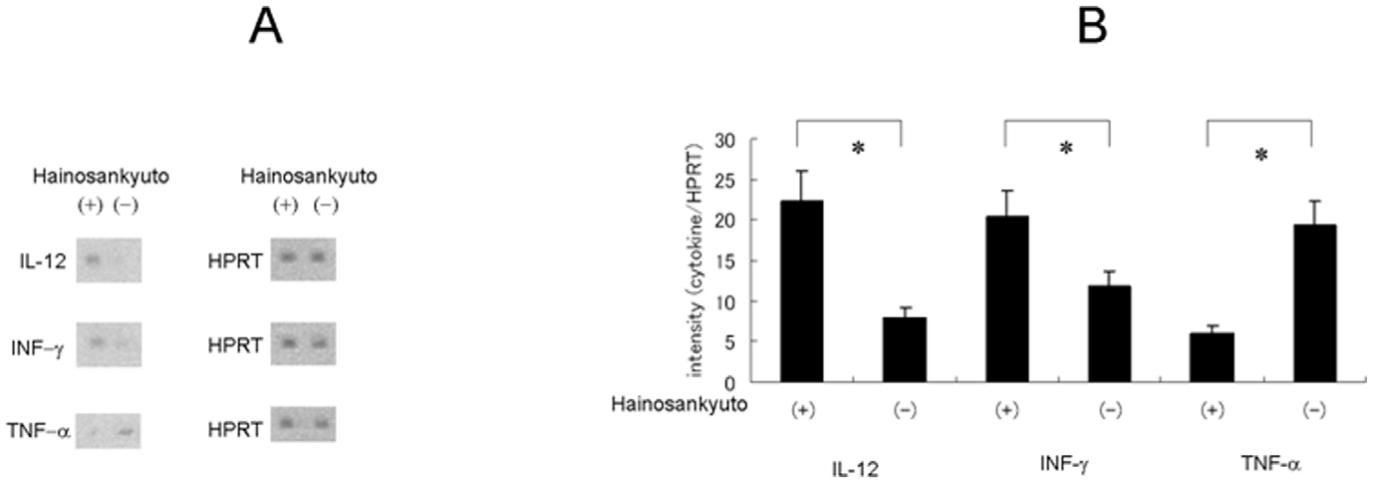
Levels of cytokine mRNA in mouse peritoneal macrophage with Hainosankyuto treatment. Three-week-old ICR mice were force-fed Hainosankyuto for 1 day. Mouse peritoneal macrophage was prepared as a single-cell suspension. Then total RNA was extracted and IL-12, IFN-γ, and TNF-α expression was detected using northern blotting analysis (Figure 8A) and quantitative real time RT-PCR (Figure 8B), with HPRT as the internal control.

We examined the cytokine expression induced by Hainosankyuto. A group of three ICR mice were force-fed Hainosankyuto and controls were force-fed PBS. After 24 hour, peritoneal macrophage was extracted from two groups of mice and three cytokine expressions were measured by northern blotting analysis (Figure 8A) and quantitative real time RT-PCR (Figure 8B). Results showed an increase in both IL-12 and INF-γ mRNA expression in mouse macrophage after Hainosankyuto treatment compared with PBS control. Conversely, the level of TNF-α mRNA expression was decreasing in mouse macrophage after Hainosankyuto treatment compared with PBS control.

## Discussion

To our knowledge, this is the first study on anti-*S. pyogenes* therapy with Hainosankyuto.

After *S. pyogenes* infection, Hainosankyuto-treated mice showed increased survival rate, blood bactericidal activity, serum levels of inflammatory cytokines including IL-12 and IFN-γ, macrophage phagocytic activity and macrophage mRNA levels of inflammatory cytokines including IL-12 and IFN-γ. These results suggest that Hainosankyuto can play an important role in protection against *S. pyogenes*.

Our data also potentially postulate that Hainosankyuto may be useful in treatment of clindamycin-resistant *S. pyogenes*. Because Hainosankyuto is not *per se* an antibiotic and has no anti-bacterial effect against *S. pyogenes*, the occurrence of Hainosankyuto-resistant *S. pyogenes* in future seems impossible. We speculate that Hainosankyuto may be efficacious when used against new strains of antibiotic-resistant *S. pyogenes*.

We also investigated the effect of Hainosankyuto against other *emm*-type *S. pyogenes* isolates (M4, M12, M28 and M49) and confirmed that Hainosankyuto had the same inhibitory effect on those isolates (data not shown). Inhibition of the growth of specific *emm*-type isolates and effects of Hainosankyuto may not be related. Our results imply that Hainosankyuto directly affects the host's immune system with regard to *S. pyogenes* infection.

Hainosankyuto is a traditional Japanese herbal medicine first prepared by Dr. Todo Yoshimasu, a 17th century Japanese medical doctor, by combining Hainoto and Hainosan, which are also traditional Japanese medicines. However, Hainosankyuto is now popular and widely prescribed for therapy in Japan. Because neither Hainoto nor Hainosan is easily available commercially, we chose Hainosankyuto for our study. Although previous studies on Hainosankyuto have not been found, one report showed the anti-inflammatory effect of both Hainoto and Hainosan [7]. Both Hainoto and Hainosan dose-dependently inhibit acetic acid-induced vascular permeability and carrageenin-induced hind-paw oedema; both features of the early exudative stage of inflammation. Each individual component of Hainosankyuto also shows anti-inflammatory effect. Platycodin D from Platycodon Root exert an anti-inflamment effect through the regulation of the NF-κ B pathway [8]. Glycyrrhizin from Glycyeehiza is a sweet triterpenoid glycoside possessing anti-inflammatory and anti-allergic activities [9], [10]. Paeoniae radix from Peony root has anti-inflammatory effects in mouse [11]. Ginger root extract, Immature Orange extract, and Jujube extract have superoxide diumutase like activities [12]. Although each component has anti-inflammation effect, this mechanism in detail has been unknown. Traditional Japanese herbal medicine is generally composed of several components and the interaction of them may enhance the effect of drugs. As further investigation from this perspective is needed, Hainosankyuto may have pronounced anti-inflammatory effects.

We tried to clarify the mechanism of this protective effect of Hainosankyuto. Our data suggest that the protection provided was not due to any direct anti-bacterial effect of Hainosankyuto. Augmentation of blood bactericidal activity might help the host eliminate bacteria at the infection site, and innate immunity is important in this elimination. IL-12-induced IFN-γ expression provided protection against *S. pyogenes* infection in a murine model by enhancing innate immunity [13], [14]. TNF-α suppression also plays a crucial protective role in severe *S. pyogenes* infection [15]. Not only blood bactericidal activity but also mouse peritoneal macrophage phagocytic activity, was enhanced in the Hainosankyuto-treated group. Hainosankyuto-treated mice showed significantly higher IL-12 and IFN-γ levels and a significantly lower TNF-α level than untreated mice. Taken together, we suggested that Hainosankyuto-induced cytokine production augments bactericidal activity including phagocytic activity which in turn causes bacterial elimination.

In addition, we next tried to clarify whether Hainosankyuto would provide protection if given only after bacterial infection. Although we did not find the significant recovery of survival rate in direct Hainosankyuto administration only after *S. pyogenes* infection, our results showed that the sizes of ulcer lesion were significant smaller after Hainosankyuto administration compared to PBS administration. Hainosankyuto may be useful for therapy of local lesion after *S. pyogenes* infection. Further clinical study involving human subjects about *S. pyogenes* therapy using Hainosankyuto is desired. Also whether a similar effect occurs in other bacterial infections warrants further investigation.

In summary, Hainosankyuto was more effective in preventing *S.pyogenes* infection than in administration only after *S. pyogenes* infection. We suggest Hainosankyuto as a candidate for novel anti-*S. pyogenes* therapy.

## Materials and Methods

### Bacterial strains

*S. pyogenes* 1529 and D2TY were clinical isolates from severe invasive disease in Japan [16], [17]. *S. pyogenes* D2TY in particular has a macrolide-resistant gene, *ermB*. A fresh colony was inoculated overnight on blood agar (Nihon Becton Dickinson, Tokyo, Japan) and cultured for 12 hour at 37°C. The bacteria were harvested by centrifugation and re-suspended in sterile PBS. Bacterial density was determined by measuring absorbance at 660 nm (A660). The bacterial suspension was then diluted with PBS to 10^9^ CFU (colony forming unit)/mL using a standard growth curve to relate measured A660 to bacterial concentration.

### Compound

Hainosankyuto was provided as a generous gift from the Tsumura Company Limited (Tokyo, Japan) (Table 1), and it was suspended in distilled water to prepare the stock solution at a concentration of 0.1 g/mL.

**Table 1:** Crude composition of hainosankyuto extract.

Crude drug	Composition (g)	Yield (g)
*Platycodon Root*	4.0	4.5
*Glycyrrhiza*	3.0	
*Immature Orange*	3.0	
*Peony Root*	3.0	
*Jujube*	3.0	
*Ginger*	1.0	

### Susceptibility testing

MIC of Hainosankyuto was determined using the micro-dilution method in accordance with the guidelines of the Clinical and Laboratory Standards Institute [18]. MIC was defined as the lowest concentration showing no visible growth after 24 hour of incubation at 35°C compared to the control.

### Murine model of invasive skin and soft tissue infection

The ability of *S. pyogenes* to cause local skin lesions in mice after subcutaneous inoculation was assessed using a procedure described elsewhere [19]. In brief, *S. pyogenes* was harvested after 16-hour growth on brain heart infusion agar (Eiken Chemical Co., Tokyo, Japan) containing 0.3% yeast extract (BHY agar) with appropriate antibiotics mixed in 1 mL of PBS and then centrifuged at 2000 *g* for 2 min. The pellets were diluted in 100 µl PBS to 1×10^8^ CFU and then injected under the skin surface of inbred 3-week-old female Slc∶ICR mice (Japan SLC, Inc., Shizuoka, Japan) using a 25-gauge needle. The number of CFU injected was verified for each experiment by plating the bacteria on BHY agar and counting CFU. Mice were observed daily for lesion and necrosis formation. Lesion sizes (length×width) were measured with length determined as the longest dimension of the lesion. In the Hainosankyuto-treated group, mice were force-fed Hainosankyuto (0.1 mL/10 g body weight (bw)) on days −1, 0, 1 and 2 after *S. pyogenes* inoculation. Mice in the control group were given an equal volume of PBS and were infected using the same method. We also performed another protocol to clarify the effects of Hainosankyuto in animals developing signs. In the Hainosankyuto-treated group, mice were force-fed Hainosankyuto (0.1 mL/10 g body weight (bw)) on days 1, 2, 3 and 4 after *S. pyogenes* inoculation. We evaluated the mortality and the size of necrosis using same methods as prophylactic assay.

### Blood bactericidal assay

Blood bactericidal assays were performed as an evaluation of whole blood bactericidal effect with some modification [19]. In the Hainosankyuto-treated group, mice were force-fed Hainosankyuto (0.1 mL/10 g body weight (bw)) on 1 day before collection. Mice in the control group were given an equal volume of PBS. Heparinised blood was collected from Hainosankyuto-treated and untreated mice not infected with *S. pyogenes*. Blood bactericidal assays were performed as follows; approximately 1000 CFU of strain was added to 1 mL of heparinized ICR mice whole blood. The samples were incubated at 37°C on a rotator. Aliquots were removed and plated on BHY agar to determine the number of CFU after 0, 30 and 60 min. Data were expressed as bacterial CFU counts at each times.

### Analysis of serum cytokines during infection

We determined the levels of serum cytokines, such as IL-12, IFN-γ and TNF-α during the course of infection in *S. pyogenes*-infected ICR mice treated with Hainosankyuto. Blood from both infected and treated/infected groups was collected under anaesthesia at 1 and 3 days after infection. Each blood sample was centrifuged, and the resultant serum was stored at −80°C until the day of assay. IL-12, IFN-γ and TNF-α levels were determined by sandwich ELISA (Quantikine, R&D Systems Inc., MN, USA). For the assay, combinations of capture and biotinylated mAb were used as recommended by the manufacturer. Cytokine levels were calculated using standard murine recombinant cytokine curves run on the same immunoplate.

### Macrophage cells

In the Hainosankyuto-treated group, mice were force-fed Hainosankyuto (0.1 mL/10 g body weight (bw)) on 1 day before peritoneal lavage. Mice in the control group were given an equal volume of PBS. Mouse peritoneal macrophage cells were harvested by lavage with 6 ml of cold Hank's balanced salt solution (Wako pure chemical industry, Osaka, Japan) from the peritoneal cavities. The cells were washed twice and suspended in culture medium to give 10^6^ cells/mL. Five hundred µL of the cell suspension was dispensed into 1.5 mL tube and incubated for 2 hour at 37°C in 5% CO_2_. The culture medium was removed gently by aspiration and changed to fresh culture medium. For cell culture, RPMI-1640 (Wako pure chemical industry) supplemented with 5% inactivated fetal bovine serum was used. All cells were suspended in the culture medium, plated onto 24-well culture plate at dose of 6×10^5^ cells per well (final volume, 0.6 mL) and cultured in a humidified chamber at 37°C with 5% CO_2_ and 95% air. The 5 hour culture was washed three times with Hanks' balanced salt solution to remove nonadherent cells and the adherent cells only were used as the macrophage culture.

### Macrophage phagocyte assay

Mouse peritoneal macrophage were mixed with *S. pyogenes* and incubated for 1 hour with shaking at 37°C. Briefly, macrophage phagocyte assays were performed using polypropylene tubes containing 100 µl mouse macrophage (2×10^5^ cells) and 100 µL *S. pyogenes* to yield a final concentration of 10^5^ CFU/mL. *S. pyogenes* was opsonized with mouse serum for 30 min before mixing. The samples were incubated at 37°C on a rotator, and aliquots were removed for quantitative and plated on BHY agar to determine the number of CFU after 0, 30 and 60 min. Data were expressed as bacterial CFU counts at each times.

### Northern blotting analysis

Total RNA was extracted using by QIAGEN RNA extraction kit (QIAGEN, Hilden, Germany) according to the manufacture's instructions. Approximately five µg of each total RNA preparation was electrophoresed on 1% agarose gels containing 1.1 M formaldehyde. The RNA was transferred to a Hybond-N+ membrane (GE Healthcare, Waukesha, WI). For DNA probes, primers used were: HPRT, 5′-GTTGGATACAGGCCAGACTTTGTTG-3′ and 5′-GAGGGTAGGCTGGCCTATAGGCT; IL12, ATGGCCATGTGGGAGCTGGAGAAAG and 5′-GTGGAGCAGCAGATGTGAGTGGCT-3′; INF-γ, 5′-AACGCTACACACTGCATCT-3′ and 5′-TGCTCATTGTAATGCTTGG-3′; TNF-α, 5′-GTTCTATGGCCCAGACCCTCACA-3′ [20]. The mRNA was detected with 32P-labeled HPRT, IL12, INF-γ, and TNF-α DNAs (random primer DNA labeling kit, version 2; Takara Bio Co. Ohtsu, Japan). The membranes were then autoradiographed and analyzed at room temperature with a bioimaging analyzer (BAS-1800II; Fujifilm, Tokyo, Japan). Triplicate assays were performed with RNA from at least three independent cultures.

### Quantitative real time RT-PCR

Five µg of RNA was reverse transcribed using SuperScript first-strand synthesis system (Invitrogen Co., Carlsbad, CA, USA). The cDNA samples were then subjected to PCR analysis. Primers used were described as above (Northen blotting analysis section). To quantitate mRNA, a real-time RT-PCR was performed by using the GeneAmp 7900 sequence detection system with SYBR green reagent (Life technologies Corp., Carlsbad, CA). Samples were subjected to 40 cycles of amplification consisting of 95°C for 15 s followed by 60°C for 1 min according to the manufacture's instructions. Each assay was normalized to HPRT mRNA.

### Statistical analysis

Comparison between groups regarding blood bactericidal and cytokine assays were made using the *t*-test. Survival data were assessed by Kaplan–Meier survival analysis and tested for significance using the log-rank test. Lesion size data were also tested for significance using the unpaired two-tailed *t*-test. A *p*<0.05 were considered significant.
